# Implementation and Evaluation of an Intervention for Children in Afghanistan at Risk for Substance Use or Actively Using Psychoactive Substances

**DOI:** 10.1155/2017/2382951

**Published:** 2017-07-05

**Authors:** Abdul Subor Momand, Elizabeth Mattfeld, Brian Morales, Manzoor Ul Haq, Thom Browne, Kevin E. O'Grady, Hendrée E. Jones

**Affiliations:** ^1^United Nations Office on Drugs and Crime, Kabul, Afghanistan; ^2^United Nations Office on Drugs and Crime, Vienna, Austria; ^3^Bureau of International Narcotics and Law Enforcement, US Department of State, Washington, DC, USA; ^4^United Nations Office on Drugs and Crime, Islamabad, Pakistan; ^5^Department of Psychology, University of Maryland, College Park, MD, USA; ^6^UNC Horizons and Department of Obstetrics and Gynecology, University of North Carolina, Chapel Hill, NC, USA; ^7^Departments of Psychiatry and Behavioral Sciences and Obstetrics and Gynecology, School of Medicine, Johns Hopkins University, Baltimore, MD, USA

## Abstract

The present study examined the impact of a novel intervention for children at risk for substance use or actively using substances that was provided to 783 children between 4 and 18 years of age in Afghanistan. They received the Child Intervention for Living Drug-free (CHILD) protocol while in outpatient or residential treatment. CHILD included age-appropriate literacy and numeracy, drug education, basic living safety, and communication and trauma coping skills. A battery of measures examined multiple child health domains at treatment's start and end and 12 weeks later. For younger children, there were no significant Gender or Gender X Time effects (all* p*'s > .16 and .35, resp.). The time main effect was significant for all outcomes (all* p*'s < .00192, the prespecified per-comparison error rate). Post hoc testing showed significant improvements from residential treatment entry to completion for all scales. For older children, a time main effect was significant for (all* p*'s < .00192, the prespecified per-comparison error rate) all but one outcome. Community follow-up means were significantly lower than residential treatment entry means. CHILD had a positive impact on children, and treatment impact endured from posttreatment to follow-up assessment.

## 1. Introduction

With an estimated population of more than 30 million inhabitants, Afghanistan is composed of more than ten ethnic and tribal groups, most of whom have lived together in the country for centuries. These include the majority Pashtuns, who constitute almost one-half of the population, followed by a quarter of the population of Tajiks (27%) and sizeable communities of Uzbecs (9%) and Hazara (9%). Turkmen (3%), Aimaq (4%), Baluch (2%), and small communities of Brahui, Nuristani, Pashaie, Pamiri, Khirgiz, and Qizilbash are also represented. Each of these groups has developed its own forms of languages, culture, and religious beliefs over the course of Afghanistan's history. However, the centuries-long interaction between all these groups, though distinguishable by accent and clothing (as examples), has resulted in a cultural blending of various Afghan ethnic and tribal traditions. The country is almost exclusively Muslim with a majority Sunni population (80%) and an estimated 19% Shi'i population. Afghan, Persian, or Dari is the official language, spoken by about one-half of the population, with Pashtu, also an official language, spoken by some 35% of the population. Turkic languages are also spoken by some groups (11%), as well as another thirty minor languages that have been identified (e.g., Baluchi and Pashai). Many individuals speak more than one language.

Afghanistan experienced a long period of relative peace until 1978. Up until that point, it was a relatively thriving and vibrant nation that provided women and children with many appropriate health and social services and freedoms. A short period of unrest and then establishment of a Soviet-allied regime followed, leading to invasion by Soviet troops in 1979. This invasion led to 10 years of armed resistance against Soviet troops, resulting in a mass exodus of approximately 25% of the total Afghan population, a majority of whom were women and children [1], followed by years of civil war among warlords for control of the country, with, first, the establishment of the Democratic Republic of Afghanistan, subsequently overthrown by the Islamic State of Afghanistan, eventually leading to Taliban rule of Afghanistan in 1997. The United States then launched “Operation Enduring Freedom” leading to the establishment of a democratic government in 2001. Since that time, Afghanistan has witnessed a fragile peace, threatened repeatedly by the Taliban and Al-Qaeda forces [2].

The result of this continuing warfare and violence in Afghanistan has been multifold. Afghanistan is now one of the poorest, most ravaged countries in the world. Figures for 2012 indicate that its population has an average life expectancy of 60.5 years, and its infant mortality rate is one of the highest in the world. The percentage of children moderately or severely underweight is 33%. Among adults living in Afghanistan, the most common stressful events experienced include lack of shelter (70%) and lack of food and water (56%). Anxiety, depressive symptoms, and posttraumatic stress disorder (PTSD) were identified in 72%, 68%, and 42% of respondents, respectively. Women were found to have worse mental health status relative to men [3]. Only 53.9% of the population has access to mobile phones and 5.5% have access to the Internet [4].

With more than 30 years of war and conflict, the educational system of Afghanistan is extremely strained. Figures from 2011 [5] indicate that attendance rates in primary and secondary school in Afghanistan are 66% and 18% for boys and 40% and 6% for girls, respectively. The literacy rate for individuals 15–24 years old is 49% for males and only 18% for females. Among all adults, the literacy rate is 43% for men and 13% for women [4]. Thus, there is a dire need for women and men of all ages to have basic education.

A survey in 2010 [6] reported that approximately 1 million (8%) Afghans 15–64 years of age are dependent on psychoactive substances, a percentage twice that of the global average [6]. The Afghanistan National Urban Drug Use Survey found that 11.4% of the population tested positive for any psychoactive substance, with opioids prevalence at 5.6%, accounting for more than 50% of substance use in women and children but only a third of substance use in men (Cottler and Colleagues) [7]. A study conducted by Todd and colleagues [8] showed that 385 out of 483 IDUs completed one or more follow-up visits. All participants were male with a median age of 28 years and a median duration of injecting of 2 years. HCV and HIV risk are high and there is a need for increasing awareness of HCV transmission and overdose risk and to prepare clients for harm reduction needs during conflict and displacement and efforts are needed to engage community and police force. The Afghanistan National Drug Use Survey (ANDUS) 2015 [9] estimated between 2 and 2.4 million individuals in Afghanistan use psychoactive substances, which is 7.3% of the population (16.1% men, 9.5% women, and 0.8% children). Afghanistan has one of the highest opiates use rates worldwide. The national adult substance use rate is 12.6%, more than double the global psychoactive substance use rate of 5.2%. Substance use is estimated to affect almost one in three households in the country. The rate of substance use in rural areas is 2.5 times that in urban areas. Children in rural areas are far more likely to test positive for substance use. The substance-positive rate among rural children is estimated at 11.3% while for urban children the rate is estimated at 2.3%. Almost 91% of children who test positive have been affected by secondary contact with smoke from substances, typically opium, smoked by adults in the home or care environment. Opioids are the most commonly used psychoactive substance.

Gupta [10] surveyed 300 children 8–18 years of age in Kabul, finding that 72% had lost a family member in the past five years, among whom 40% had been a parent. Being a witness to acts of violence and death was almost universal in the sample. Almost 75% believe they would not live into adulthood, and more than 50% could be seen to have psychological problems that affected their childhood activities. Catani [11] and colleagues reported on schoolchildren 7–15 years of age in Kabul (122 girls, 165 boys). Traumatic events, particularly violence in the home, were experienced more frequently by boys than by girls. Probably posttraumatic stress disorder was found in 26% of boys and 14% of girls. Child labor was a frequent occurrence and was a risk factor for maltreatment of girls at home. Like their adult counterparts, girls are more likely than boys to suffer mental health problems and have depressive symptoms [12].

The literature regarding the state of affairs in Afghanistan illustrates several important themes that serve as the basis for an intervention targeting children. These themes include opium use, lack of formal education, lack of basic health education, high rates exposure to stressful living situations, violence and trauma, and related comorbid mental health problems. The Child Intervention for Living Drug-free (CHILD) Protocol [formerly known as the Child Addiction Treatment (CAT) protocol] (described in detail, below) is a novel, culturally sensitive psychosocial intervention designed to address the multidimensional nature of problems faced by these children in a residential setting that also provided supportive services for the children. CHILD was not a research protocol; rather, it was an empirically based intervention developed to meet the needs of substance-using children in Afghanistan. However, it collected outreach, pretreatment, posttreatment, and follow-up data to inform the treatment staff of progress of each child during and after treatment.

The purpose of the present study is twofold: (1) describing the implementation of the CHILD intervention protocol and (2) reporting preliminary outcome data in regard to its impact.

## 2. Methods

### 2.1. Institutional Review Board (IRB) Approval

The study protocol, including informed consent procedures, was reviewed and approved by the Johns Hopkins University Institutional Review Board as well as the Ministry of Public Health of Afghanistan's Institutional Review Board.

### 2.2. Informed Consent

All participants and/or their legal guardian(s) provided written informed consent to take part in the study. The aims of the study were explained and participants were informed that they could withdraw at any time without any further obligation to provide data, and they would continue in residential treatment but would not be provided with the CHILD treatment components. Consent was read to the parent(s)/guardian and if they could not write, they applied a thumb print to stamp the consent. In addition, during outreach the outreach team used assent (oral consent) because in outreach it was very difficult to obtain written consent based on the general Afghans culture beliefs and practices to such a request as well as the low literacy rate.

### 2.3. Participants

Participants were 699 children (373 girls, 326 boys), 4–7 years of age, and 84 older children (1 girl, 83 boys) 8–18 years of age. No additional demographic information was collected from the children.

The data on the children reported in this paper is for the first 783 children who came in contact with the CHILD program and were among the first of several thousand children who were screened by the program and entered treatment. Moreover, of these participants, 144 younger children were screened and went directly to residential treatment, with no outpatient period, and 8 directly entered residential treatment without screening, while 30 older children entered outpatient treatment without screening. These variations to the screening protocol were largely based on treatment need.

### 2.4. Procedures

The Child Intervention for Living Drug-free (CHILD) protocol was implemented in the Kabul, Herat, Balkh, Nangarhar, and Badakhshan provinces of Afghanistan. The project has three interconnected components: outreach services, outpatient services, and residential substance use treatment. There were 12 outreach teams, 10 outpatient centers, and 10 substance use treatment centers, with a total of 325 beds (120 beds for children, 70 beds for women, 110 beds for male adolescents, and 25 beds for female adolescents). Treatment duration for residential treatment was 45 days for children and 180 days for older children.

Outreach activities involved trained staff circulating in the capital city of each province as well as nearby secure districts to identify children who were at risk of substance use or actively using substances. Outreach staff did not leave the secure areas for safety reasons. Outreach staff approached children under one of three different scenarios. In the first scenario, they approached a family with a child who had been identified as using illicit substances by a community leader; in the second, they approached children directly on the street; in the third, they approached children in orphanages. Each outreach team used a screener form (see Measures). A respondent who scored positive (one or more true responses) in any of the above five screening areas was referred to an outpatient center for assessment.

Trained staff in the outpatient centers assessed the children with a battery of assessment instruments (see Measures). A child positive for substance use was referred to a residential center for treatment, while a child negative for substance use but deemed at risk for such use would visit an outpatient center every day for eight weeks to receive a comprehensive psychosocial intervention that included education, life skills, and individual counseling. Outpatient centers also provided treatment for minor ailments as well as lunch and snacks. A child with severe medical or psychological problem was referred for appropriate services in the community.

Children who were deemed to be at risk for psychoactive substance use or were actively using such substances were referred to residential centers, where they were assessed prior to treatment with the same measures as used in outpatient center assessments to develop a treatment plan. If a child had a family member who was using psychoactive substance(s), then staff considered the child as at risk, while if the child himself or parent(s) were reporting that the child was actively using psychoactive substances then s/he was considered using a psychoactive substance. The length of time between outpatient and residential intake assessment was variable and dependent on when the child appeared at the residential treatment center but typically was between 2 and 7 days. Upon completion of treatment, the patients were again assessed to evaluate the treatment progress.

After discharge from the residential treatment center the child or adolescent was followed in the community on a weekly basis by an outreach team. At the end of 12 weeks in the community, outreach staff conducted a reassessment to determine the need for further services or to determine that the weekly visits could be terminated.

### 2.5. Outreach Staff: Training and Experience

Outreach staff were experienced social workers and psychologists trained both in how to carryout outreach activities and in the CHILD protocol.

### 2.6. Assessment Staff: Training and Experience

Outpatient staff were psychologists, social workers, and a medical doctor, all of whom were trained in how to conducted assessment activities and in the CHILD protocol.

### 2.7. Interventionist Staff: Training and Experience

Interventionist staff were members of a multidisciplinary team consisting of experienced psychologists, social workers, nurses, and medical doctors, all of whom were trained on the CHILD protocol.

### 2.8. Intervention: Child Intervention for Living Drug-Free (CHILD) Protocol

CHILD is a comprehensive psychosocial intervention built on multiple prevention and treatment platforms and so employs motivational interviewing techniques, contingency management, skill-building education, traditional education, trauma-informed care, and art therapy techniques. It was developed for use with children either at risk for or actively using psychoactive substances.

The CHILD psychosocial program was provided 5 times a week to participants whom screening indicated were at risk for or actively using psychoactive substances while they were inpatient (45 days for children and 180 days for adolescents). Each group session lasted approximately 1 hour. An example schedule of inpatient components of treatment for the younger children can be found in Table 1. The older children schedule repeated this 6-week cycle three additional times, during which time the material covered included past material that was both reinforced and new material that was introduced.

The components of the CHILD protocol were as follows.

*Age-Appropriate Basic Education*. For younger children, the foundation of education is covered thrice weekly. Examples of topics covered include the alphabet, shapes, size relationships, opposites, writing of letters and numbers, and numbers and numeric relationships. For older children, literacy and basic mathematic functions, applying math to daily living, are covered. 

*Drug Education*. In an age-appropriate manner, children were taught that drugs change the way your body and mind work. Some medicines are helpful when used in the right way. Helpful and harmful drugs are explained. How drugs cause illness, impaired coordination, slowed growth, and emotional harm such as feelings of isolation or paranoia is discussed, with a focus on developing life skills such as refusal skills and decision making skills. The legal issues associated with drug and alcohol use are treated, because a conviction for a drug offense can lead to prison, loss of a job education. Talk about positive, drug-free alternatives forms the basis of the lesson, and they are explored with the counselor. Discussions are focused on providing information about the harmful use of drugs, without using judgmental terms and without creating a sense of fear about drug use. 

*Nutrition*. Using the UNICEF publications, the nutritional information is tailored to the age of the children. Topics covered include a discussion of what are healthy foods and the types of vitamins and minerals needed for healthy growth, and how to prepare healthy foods and simple meals is discussed. 

*Hygiene*. In an age-appropriate manner, the importance of hand-washing, brushing teeth, and caring for the body is discussed. The use of toilets or latrines and the need to practice good hygiene, protect water sources, and safely dispose of waste water and refuse are presented. Children are taught how to make soap. 

*Personal Safety*. In an age-appropriate manner, topics covered include basic living safety such as avoiding open cooking fires, bare light bulbs, live electrical wires, land mines, identifying hazards in the home, basic ways to stay healthy, and how to interact with adults and avoid and/or keep themselves safe during family violence situations. 

*Trauma Coping Skills*. Participants are taught to deal with intrusive thoughts and feelings, skills to reduce arousal (relaxing, concentrating, and sleeping), and skills to manage avoidance (fears/difficulties facing reminders). Role-playing and practices of skills are undertaken. Participants draw, write, and talk about the incidents. They are shown how to look to the future rather than the past (avoidance). 

*Communication Skills*. In an age-appropriate manner, effective communication techniques are taught. Items covered include interaction and communication with adults, conflict resolution, positive self-talk, culturally appropriate self-advocacy communication, and deescalation of angry situations. 

*Structured Art Expression Techniques*. In an age-appropriate manner, participants are given materials and guided to explore and express emotions. Topics include making self-masks, memory boxes, boxes representing themselves, painting pictures to express emotions, and creating clay objects to express stories about their life events.

### 2.9. Measures

Four measures (SDQ, CRIES, ASCL, and SRQ-20) are already available in Afghanistan in Dari and Pashto versions; two (SCARED and QOL) were translated into Dari/Pashto and fully backtranslated into English by members of the research team who were fluent in both Dari/Pashtu and English and then modified as necessary to permit culturally sensitive administration of the items. Although all measures were originally developed as self-report instruments, because of the low literacy rate in the country and very low literacy rate among the substance-using child population all instruments were administered by clinic staff who had been trained in their administration. Administration was in the language chosen by the child.

Measures were chosen to be age-appropriate, so that there was one set of measures for younger children and another set for older children. Only one measure was in common to both groups, other than the screening instrument.

#### 2.9.1. Measures Administered to Both Younger and Older Children

*Screening Form (SF)*. The SF consisted of 16 3-point questions (e.g., 0 = “None”; 1 = “Some”; 2 = “A lot”) that screened for problems in five areas: behavioral, emotional, and social problems; psychological distress; trauma exposure; physical health/medical problems; and substance use. A respondent who scored above 0 in any of the above five areas was referred to an outpatient center for assessment. 

*Child Strengths and Difficulties Questionnaire (SDQ)*. The SDQ [13, 14] is an internationally validated 25-item questionnaire providing balanced coverage of behavioral, emotional, and social problems that asks the child about “your behavior over the past 6 months.” Items are scored 0, 1, or 2 (with some items reverse-scored). The SDQ has 5 scales of 5 items each: Emotional Symptoms (ES), Conduct Problems (CP), Hyperactivity (H), Peer Problems (PP), and Prosocial (P) scales. There is a separate score for each scale. There is also a total difficulties' score, which is ES + CP + H + PP scores (omitting the Prosocial subscale).

#### 2.9.2. Measures Administered to Younger Children

*Child Revised Impact of Events Scale (CRIES)*. The CRIES [15–18] is a screening tool measuring risk for child posttraumatic stress symptoms. It has 8 questions that ask the child to respond on 4-point scale with “Not at all” = 0, to “Often” = 3. It has shown good internal consistency reliability (Cronbach's *α* = 0.82) and seven-day test-retest reliability (*r* = 0.78, *p* < .0001) in research in Afghanistan. 

*Self-Report for Childhood Anxiety Related Emotional Disorders (SCARED)*. The SCARED [19] is a reliable and valid screening tool for determining anxiety disorders in children and adolescents. It has 41 items that ask the child to indicate feelings for* the last 3 months*. Items are scored “Not true” = 0, “Sometimes true” = 1, or “Very true” = 2. There are 5 subscales: a 13-item Panic Disorder (P), a 9-item Generalized Anxiety Disorder (GA), an 8-item Separation Anxiety Disorder (SeA), a 7-item Social Anxiety Disorder (SoA), and a 4-item Significant School Avoidance (SS). There is also a total SCARED score, which is P + GA + SeA + SoA + SS.

#### 2.9.3. Measures Administered to Older Children

*Afghan Symptom Checklist (ASCL)*. The ASCL [20] is a 22-item check list that yields a single score with excellent reliability (*α* = .93) and good construct validity, correlating strongly with a measure of exposure to war-related violence and loss (*r* = .70). 

*Self-Reporting Questionnaire-20 (SRQ-20)*. The SRQ-20 [19, 21] was developed by the WHO to screen for psychiatric disturbance in individuals in developing countries. It has adequate reliability and internal consistency. The SRQ-20 [21] has 20 items that ask about problems “bothering you the* last 30 days.*” Items are scored “No” = 0 or “Yes” = 1. 

*Quality of Life (QOL)*. This measure was developed specifically for the Child Addiction Treatment project in Afghanistan with questions drawn from other existing quality of life measures and tailored to the Afghan culture. It asks the respondent to indicate “In the* past 3 months*, how often have you experienced…” problem severity in 20 areas of functioning. Items are scored “Never or Almost Never” = 1, to “Always or Almost Always” = 5. It has 4 subscales: a 6-item Physical Health (PH), a 5-item Mental Health (MH), a 5-item Friends (F), and a 4-item Home (H) subscales. There is a separate score for each subscale. There is also a total QOL score, which is PH + MH + F + H scores.

### 2.10. Data Entry and Management

A Microsoft Access database was written and developed to enter and store all data. Staff at the health facilities level were trained in how to enter data and use the database. All data were double-checked at the central hub data center in Kabul.

### 2.11. Statistical Analysis

Because the length of residential treatment was different for the younger and older children, analyses were conducted separately for each group.

A general linear mixed model (GLMM) analysis was conducted on the scores on the scales and/or subscales of the above-named measures, as appropriate to the measure, with all scale and subscale scores assumed to follow a normal distribution in the population. For analysis of the child data, there were three effects in the model: gender as the fixed between-subjects factor, time (outreach, residential intake, posttreatment, and follow-up) as the fixed repeated factor, and their interaction; for the adolescent data, the one female observation was omitted, and so there was a single effect: time (outreach, residential intake, posttreatment, and follow-up) as the fixed repeated factor. A familywise error rate was used to set *α* = .00192 (.05/26), where the nominal Type I error rate was set at .05, and the family included both the 13 assessment scales/subscales for the younger children (CRIES total score, the 5 subscale scores and the SCARED total score, and the 5 subscale scores and the SDQ total score) and the 13 assessment scales/subscales for the older children (ASCL total score, SRQ total score, the 5 subscale scores and the SDQ total score, and the 4 subscale scores and the QOL total score). Post hoc testing of simple mean differences associated with significant effects utilized the Dunn-Sidak multiple comparison test to control the post hoc testing error rate to at most the familywise error rate [22]. All analyses were performed by SAS version 9.3 [23].

## 3. Results

### 3.1. Feasibility and Acceptance

Individual interviews with project staff and participants showed high rates of participation and high levels of satisfaction (both over 90%) from participants and staff. Although each of the education, life skills, and one-to-one sessions was scheduled for a 60-minute time slot, some sessions consistently ran over the allotted time because children were particularly engaged. Comments about the intervention were positive (e.g., I liked the art and games; I always learn something). Overall, staff members also reported a high level of satisfaction with the intervention and expressed the need for continued implementation.

### 3.2. Follow-Up Rates

The treatment completion rate (residential intake assessment to posttreatment assessment) was 88% for younger children and 98% for older children, both outstanding considering the war-torn intervention setting and the fact that treatment dropout rates reported by inpatient drug studies have ranged from 19% to 63% [24, 25] for US adult patients in intensive inpatient programs in shorter treatment (21–28 days). Follow-up rates from posttreatment to follow-up assessments were disappointing—14% for younger children and 51% for older children. The low follow-up rate was due to a lack of emphasis by the supervisory staff in follow-up data collection and the staff were not paid to complete such visits. It was voluntary for staff to visit children in their homes during the follow-up phase and that practice proved impractical.

### 3.3. Measure Reliability

Internal consistency reliability Cronbach's *α* was outstanding for all scales (see Tables 2 and 3) and, for those scales with subscales, generally good to outstanding for the respective subscales, with the exception of the SDQ Peer Problems subscale for children.

### 3.4. Change Over Time: Younger Children

There were no significant Gender or Gender X Time effects (all* p'*s > .16 and .35, resp.). The time main effect was significant for all outcomes (all* p'*s < the per-comparison error rate of .00192) [see means (Standard Errors) in Table 2]. Post hoc testing indicated that there were significant mean decreases from residential treatment entry to residential treatment completion for all scales (the SDQ Prosocial subscale significantly increasing), with the smallest percentage change of 49%. The SCARED Generalized Anxiety Disorder and School Avoidance subscales and the SDQ Emotional Symptoms, Conduct Problems, and Hyperactivity subscales and the SDQ total mean scores significantly increased from residential treatment completion to community follow-up total score, with the SDQ Prosocial subscale mean significantly decreasing. However, with the exception of the CRIES and the SDQ Prosocial subscale means, all scales and subscale means were significantly lower at community follow-up than at residential treatment entry.

### 3.5. Change Over Time: Older Children

The time main effect was significant for all outcomes (all* p'*s < the per-comparison error rate of .00192) except for the QOL Home subscale (*p* = .33) [see means (Standard Errors) in Table 3]. Post hoc testing indicated that change from residential treatment entry to residential treatment completion was significant for all outcomes, with reductions in means for all scales except that the SDQ Prosocial mean scores significantly increased during that period. As with the results for the children, these changes were generally quite large, with 8 mean differences exceeding 50% of the baseline mean and the smallest percentage change in means, for the QOL Friends subscale, of 24%. There were no significant changes from residential treatment completion to community follow-up, with the exception of the SDQ Prosocial subscale, for which the mean score was significantly lower at community follow-up. Moreover, all community follow-up means were significantly lower than their corresponding residential treatment entry means.

### 3.6. Success Stories

In order to provide greater context to the impact of the intervention, we asked 3 interventionist staff to provide brief summaries of their contact with a child, including information about the presenting problem, the impact of the CHILD intervention, and the outcome of treatment (The name of each child given below is a pseudonym, to protect the identity of the child.)

#### 3.6.1. Ahmad

Ahmad was an innocent 14-year-old boy, born into a poor family. His father was dependent on drugs and Ahmad was always dreaming of life without financial problems like other kids. Every night he was dreaming for a better tomorrow, which never came. Ahmad wishes if his father gets treatment but unfortunately his father was not ready for the treatment. Ahmad's mother requests him to work and contribute to the family. Ahmad searched for a job but could not get one. Then he joined a group of peers who were smoking cigarettes and using hashish, opium, heroin, and alcohol for the last two years. Ahmad also used these substances. To find money to fund his substance use and support his family, Ahmad began commercial sex work. However, he felt ashamed and depressed for what he was doing, yet he had no other opportunities for income. Thus, Ahmad was open to a helping hand to move him out of his situation. Our project outreach team found Ahmad and they talked with him about the children substance use treatment facilities in Kabul, which he indicated he was willing to enter. He received treatment in a residential facility where he was provided with the CHILD intervention. He very much liked the child modules of the CHILD interventions such as art therapy, personal safety, and drug education. At the conclusion of his treatment he stopped using psychoactive substances and he returned to his family and is more hopeful for future.

#### 3.6.2. Aziz

Aziz was approximately 9 years old and was unaware that he would face problems due to drug use disorders. Aziz was the only son in his family and his parents were looking for and expecting a bright future for him. In fact, they placed all their hopes and dreams in him.

But poverty had other ideas, not allowing Aziz's parents to see his bright future comes to pass and destroying their own future ambitions. Aziz was not able to tolerate his family problems and economic fortunes. He had five sisters, who seemed satisfied with what their father was able to bring them to the family. They seemed to totally overlook their future hopes, ambitions, and wishes.

On the other hand, Aziz was neither satisfied nor pleased with the hardship and poverty of the family. He never valued his family life and was always thinking of ways to earn money and live a better life. When he turned seven, he left home and lived with his peers in the streets. Later he started working in bus stations as a conductor. Gradually he realized his hopes and dreams were gone.

He joined another group of youngsters who were pickpockets. During this time, he was exposed to psychoactive such as heroin, hashish, and wine. He spent two years with this group using various illicit substances. Finally, the outreach team found him on the street and convinced him to stop using. He participated in regular meetings before going to outpatient services, where he received psychosocial counseling, opportunities for hygiene care, and social services for about one month. The outreach team contacted his family several times to get their consent for Aziz to participate in a residential drug treatment program.

Finally both the family and Aziz gave consent, and he was placed in a residential program. He spent 45 days in the residential treatment program receiving psychosocial support through implementation of the CHILD protocols. The outreach team visited him regularly to provide support and prepare him for the transition back to the community.

After completing his residential program in residential setting, he is a totally substance-free boy. Both the outpatient and treatment programs helped him to reconnect with his family. Aziz and his family appreciated the importance of the CHILD program to Aziz and the family. Aziz is currently maintaining his substance-free status, he is happy, he continues to work, and he helps to support his family. Success for Aziz means having not only a substance-free but also a productive life.

#### 3.6.3. Farid

One day our outreach team saw a weak, skinny 13-year-old boy among other adults and children in a “drug hotspot.” He had heroin over cigarette paper and a lighter under the heroin paper. The outreach team arrived on the scene and found that he was sweaty, senseless, and laying down on the ground and the team feared that he might be on the verge of death. The outreach team transported him to an outpatient center. When he awoke he was very hungry, and he ate, took a bath, and changed into clean clothes, and he looked much better. He then started crying and weeping, indicating that this was the first time anyone had showed him such love and empathy. During his outpatient visit our staff found that he had no home and was living under a bridge during very cold winter. After his basic needs were met, he was referred to a treatment center. On intake, Farid indicated he was living in Kabul. He reported that his father said at 6 months of age his mother died, and so his aunt cared for him for three years. His father had remarried after one year of Farid's mother's death. Due to a weak economy condition his aunt and her family immigrated to Iran and returned Farid to his father and stepmother. Farid indicated he wanted to go to school but his stepmother forbade this. She would “always” beat, abuse, and insult him. She would give him a bucket to sell water. When Farid gave her “much” money she was happy; otherwise she beat him. He saw other children that they were laughing and playing and wearing new clothes. He became hopeless and wished his mother was alive to love and care for him. He had a stepbrother, and his stepmother loved him and bought him new clothes. Farid said he loved him too, but his stepmother would not let him play with his stepbrother. One day his stepmother told his father either she or Farid can live in this house. Farid's father cast him out of the house. Farid awoke crying at which point he met a man and he asked Farid to work in the man's hotel for three meals a day and spending money if he needed it. Farid washed dishes and cleaned tables and reported he was very happy with free time at night to go outside. Farid reported that one day he met three boys with red eyes who gave him a paste that was “smooth and with black color.” Farid used it and liked it. Some time later he met the boys who told him he would now need to pay for his opium. He would steal money to pay for his opium but was caught by the owner who dismissed him. Farid's life “went to darkness,” and he found himself living in dirty places and under bridges. After completion of CHILD treatment at children inpatient treatment facility, Farid was reunited with his father. Farid's father told Farid that he is happy that he found his son after such a long time. Farid was able to go home and play with his brother and to attend school.

## 4. Discussion

Findings strongly suggest that use of the assessment measures with children in Afghanistan yielded reliable scores on the constructs being measured. This inference is supported by the generally excellent internal consistency of the scales and subscales, suggesting the measures were highly reliable, and the consistency in mean changes in scores, suggesting the measures were sensitive to change in the participants.

Results indicate that both younger and older children entering treatment had widespread psychological and social problems that could be considered serious. Scores on the Afghan Symptom Checklist suggest serious symptomatology relative to previous research with Afghan students 11–16 years of age, whose mean score on the ASCL was 1 standard deviation lower than the mean score of the present sample of older children at residential treatment entry [12]. The mean on the SRQ-20 at residential treatment entry among male older children, 13.6 out of a possible 20 maximum, exceeds cutoffs of 7–10 that have been found to be indicative of psychiatric comorbidity in samples from other countries. Moreover, this mean is more than 2 standard deviations higher than the mean reported by Panter-Brick et al. [12] for their sample of Afghan students 11–16 years of age. Finally, QOL scores, transforming each subscale and the total scale score into scale means rather than scale sums (as found in Table 3), would equate to “sometimes” (3 out of 5 on the Likert response scale) in terms of average problem ratings, strongly suggesting a pervasive negative quality of life.

Third, our findings suggest that CHILD had a positive impact on those children who were assessed at posttreatment, and, for the smaller subsamples of children assessed at follow-up, impact of treatment endured from posttreatment to follow-up assessment. The CHILD intervention can be viewed as having had a strong impact during the course of residential treatment. However, results for the CRIES suggest that, given the severity of trauma in Afghan society, 45-day residential treatment for younger children may not provide sufficient time to produce improvements in child posttraumatic stress symptoms. As such, it may be important that trauma coping skills for children be continued in the outpatient and aftercare phases following 45-day residential treatment. Nonetheless, it cannot be discounted that the positive impact of CHILD could be due to a more general positive response to treatment impact rather than the specific impact of the CHILD intervention. This positive response may be a result of being given the opportunity to live without exposure to psychoactive substances and reside in a place that is physically and emotionally safe. And, despite the small sample sizes at follow-up, there is a clear and consistent suggestion of enduring impact, at least for some children. There were several limitations to the present study. The major limitation was that the CHILD treatment was not administered as part of a research project. Rather, the CHILD protocol was designed to meet the treatment needs of children, based on a needs' assessment. An assessment component was built into the CHILD protocol to guide treatment planning. The analysis of these assessment measures shed light on the treatment needs of children in Afghanistan and the promise of the CHILD protocol at addressing their needs for treatment of substance use. A corollary limitation is that there are no data describing the children or their families, due to the fact that recording such data was deemed not cost-effective to the project. A related corollary is that the project was necessarily a single-group study that focused on change within a treated group, and there is neither a control group nor, given the instability and extreme poverty in the country, the ability to identify any comparison group. However, it should be noted that the CHILD treatment protocol was developed because treatment programs already in place in Afghanistan for children had largely been unsuccessful in bringing about change.

## 5. Conclusions

Findings suggest that the CHILD intervention shows promise of producing significant change in deprived and traumatized children who are at risk for or are in need of treatment for psychoactive substance use. Results indicate that the CHILD intervention was both broadly impactful and perhaps enduring. A systematic, longer-term evaluation of the CHILD intervention compared to residential usual care is needed.

## Figures and Tables

**Table 1:** Example schedule of intervention components that younger children receive while in residential inpatient treatment for 45 days.

Week	Sunday	Monday	Tuesday	Wednesday	Thursday
1	(i) Appropriate basic education (ii) Trauma coping skills	(i) Nutrition (ii) Drug education	(i) Appropriate basic education (ii) Structured art therapy	(i) Hygiene (ii) Personal safety	(i) Appropriate basic education (ii) Communication skills
2	(i) Appropriate basic education (ii) Trauma coping skills	(i) Nutrition (ii) Drug education	(i) Appropriate basic education (ii) Structured art therapy	(i) Hygiene (ii) Personal safety	(i) Appropriate basic education (ii) Communication skills
3	(i) Appropriate basic education (ii) Trauma coping skills	(i) Nutrition (ii) Drug education	(i) Appropriate basic education (ii) Structured art therapy	(i) Hygiene (ii) Personal safety	(i) Appropriate basic education (ii) Communication skills
4	(i) Appropriate basic education (ii) Trauma coping skills	(i) Nutrition (ii) Drug education	(i) Appropriate basic education (ii) Structured art therapy	(i) Hygiene (ii) Personal safety	(i) Appropriate basic education (ii) Communication skills
5	(i) Appropriate basic education (ii) Trauma coping skills	(i) Nutrition (ii) Drug education	(i) Appropriate basic education (ii) Structured art therapy	(i) Hygiene (ii) Personal safety	(i) Appropriate basic education (ii) Communication skills
6	(i) Appropriate basic education (ii) Trauma coping skills	(i) Nutrition (ii) Drug education	(i) Appropriate basic education (ii) Structured art therapy	(i) Hygiene (ii) Personal safety	(i) Appropriate basic education (ii) Communication skills

*Notes.* The first three days of inpatient stay would involve baseline assessment, introduction to the residential rules and code of conduct, and the like. Older children would cycle through these same components three additional times during their residential stay—some basic material would repeat, as necessary (e.g., education, drug education) while most components would involve additional material.

**Table 2:** Internal consistency reliability at residential treatment entry, estimated marginal means (M), Standard Errors (SE), and sample sizes [*n*] at outpatient assessment, residential treatment entry, residential treatment completion, and community follow-up for the younger child sample (*N* = 689).

Evaluation measure	*α*	Outpatient assessment	Residential treatment entry	Residential treatment completion	Community follow-up
		M (SE) [*n*]	M (SE) [*n*]	M (SE) [*n*]	M (SE) [*n*]
Child Revised Impact of Events Scale (CRIES)	.95	^a^9.6 (.29) [544]	^b^5.5 (.26) [689]	^c^1.7 (.27) [607]	^a,d^10.5 (.72) [83]

Self-Report for Childhood Anxiety Related Emotional Disorders (SCARED)					
Panic Disorder	.94	^a^14.2 (.28) [544]	^b^15.6 (.25) [686]	^c^2.8 (.26) [604]	^c^4.1 (.71) [82]
Generalized Anxiety Disorder	.91	^a^8.6 (.20) [544]	^b^9.6 (.18) [686]	^c^2.0 (.19) [604]	^d^4.4 (.52) [82]
Separation Anxiety Disorder	.88	^a^9.2 (.17) [544]	^a,b^9.8 (.15) [686]	^c^3.5 (.16) [604]	^c^4.1 (.43) [82]
Social Anxiety Disorder	.83	^a^7.9 (.14) [544]	^b^8.6 (.13) [686]	^c^3.6 (.13) [604]	^c^4.6 (.36) [82]
School Avoidance	.88	^a^3.1 (.11) [544]	^b^4.2 (.09) [686]	^c^0.6 (.10) [604]	^d^1.6 (.27) [82]

Total SCARED Score	.95	^a^43.1 (.79) [544]	^b^47.9 (.70) [686]	^c^12.5 (.75) [604]	^c^18.3 (2.03) [82]

Child Strengths and Difficulties Questionnaire (SDQ)					
Emotional Symptoms	.81	^a^5.4 (.11) [538]	^a,b^5.7 (.10) [685]	^c^1.3 (.11) [609]	^d^2.6 (.29) [83]
Conduct Problems	.75	^a^4.9 (.10) [538]	^b^5.3 (.09) [685]	^c^1.5 (.10) [609]	^d^2.9 (.27) [83]
Hyperactivity	.70	^a^5.02 (.10) [538]	^b^6.0 (.09) [685]	^c^2.4 (.10) [609]	^a^4.3 (.26) [83]
Peer Problems	.56	^a^4.9 (.10) [538]	^a,b^4.9 (.09) [685]	^c^2.5 (.09) [609]	^c^3.2 (.25) [83]
Prosocial	.74	^a^4.8 (.12) [538]	^b^3.3 (.11) [685]	^c^6.3 (.12) [609]	^b^3.3 (.31) [83]

Total SDQ Score	.91	^a^20.3 (.36) [538]	^b^21.9 (.32) [685]	^c^7.8 (.34) [609]	^d^12.9 (.92) [83]

*Notes*. *α* = Cronbach's measure of internal consistency reliability, which was calculated at residential treatment entry (at the largest sample sizes). Theoretical range of scores—CRIES: 0–24; SCARED Panic Disorder subscale: 0–26; SCARED Generalized Anxiety Disorder subscale: 0–18; SCARED Separation Anxiety subscale: 0–16; SCARED Social Anxiety subscale: 0–14; SCARED School Avoidance subscale: 0–8; SCARED Total scale: 0–82; SDQ subscales: 0–10; SDQ Total scale: 0–50. For all measures except the SDQ Prosocial subscale, higher scores indicate *greater* problem severity in the area measured (for the SDQ Prosocial scale, higher scores indicate a more prosocial orientation). Sample sizes do not equal 689 because not all children were given all measures at all times. A model that included the Gender X Time interaction effect for both SCARED Panic Disorder and SDQ Hyperactivity failed to reach a solution due to an infinite likelihood, so the model was refit without the interaction term; the means in this table were estimated from this latter model. Means that share the same superscript are not significantly different from each other using the Dunn-Sidak correction to conduct post hoc tests.

**Table 3:** Internal consistency reliability at residential treatment entry, estimated marginal means (M), Standard Errors (SE), and sample sizes [*n*] at outpatient assessment, residential treatment entry, residential treatment completion, and community follow-up for the older male sample (*N* = 84).

Evaluation Measure	*α*	Outpatient entry	Residential treatment entry	Residential treatment completion	Community follow-up
		M (SE) [*n*]	M (SE) [*n*]	M (SE) [*n*]	M (SE) [*n*]
Afghan Symptom Checklist (ASCL)	.96	^a^61.4 (1.59) [83]	^a,b^51.6 (1.59) [83]	^c^25.5 (1.63) [79]	^c^27.4 (2.23) [41]

Self-Reporting Questionnaire-20 (SRQ-20)	.93	^a^15.9 (.56) [84]	^a^13.6 (.57) [81]	^c^1.8 (.58) [79]	^c^1.8 (.80) [41]

Child Strengths and Difficulties Questionnaire (SDQ)					
Emotional Symptoms	.85	^a^2.81 (.28) [84]	^b^4.5 (.28) [82]	^c^0.6 (.29) [78]	^c^1.3 (.37) [41]
Conduct Problems	.92	^a^2.6 (.32) [84]	^b^5.1 (.32) [82]	^c^0.4 (.33) [78]	^a,c^1.1 (.44) [41]
Hyperactivity	.90	^a^2.9 (31) [84]	^b^5.3 (.32) [82]	^c^0.5 (.32) [78]	^c^1.2 (.43) [41]
Peer Problems	.78	^a^2.6 (.26) [84]	^b^3.8 (.26) [82]	^a,c^2.0 (.26) [78]	^c^1.4 (.33) [41]
Prosocial	.74	^a^2.7 (.32) [84]	^a^2.6 (.32) [82]	^c^6.3 (.33) [78]	^d^0.8 (.44) [41]

Total SDQ Score	.97	^a^10.9 (1.10) [84]	^b^18.7 (1.11) [82]	^c^3.6 (1.13) [78]	^c^5.3 (1.46) [41]

Quality of Life scale (QOL)					
Physical Health	.93	^a^19.2 (.57) [84]	^a^18.4 (.58) [82]	^c^8.3 (.59) [78]	^c^8.6 (.81) [41]
Mental Health	.84	^a^15.2 (.39) [84]	^a^13.6 (.39) [82]	^c^9.3 (.40) [78]	^c^8.2 (.55) [41]
Friends	.82	^a^14.1 (.38) [84]	^a^13.8 (.39) [82]	^c^10.5 (.40) [78]	^c^9.2 (.55) [41]
Home	.72	6.9 (.27) [84]	6.7 (.27) [82]	7.3 (.28) [78]	7.1 (.37) [41]

Total QOL score	.92	^a^55.4 (1.32) [84]	^a^52.1 (1.34) [82]	^c^35.3 (1.37) [78]	^c^32.9 (1.89) [41]

*Notes*. *α* = Cronbach's measure of internal consistency reliability, which was calculated at residential treatment entry. Theoretical range of scores—ASCL: 22–110; SRQ-20: 0–20; SDQ subscales: 0–10; SDQ Total scale: 0–50; QOL Physical Health subscale: 6–30; QOL Mental Health and Friends subscales: 5–25; QOL Home 4–20; QOL Total score: 20–100. For all measures except the SDQ Prosocial subscale, higher scores indicate *greater* problem severity in the area measured (for the SDQ Prosocial scale, higher scores indicate a more prosocial orientation). Sample sizes do not equal 84 because not all boys were given all measures at all times. Means that share the same superscript are not significantly different from each other using the Dunn-Sidak correction to conduct post hoc tests. Post hoc testing among the means was not conducted for the QOL Home subscale, as the test of the time effect was not significant for this outcome.
